# Impact of COVID-19 pandemic on residency and fellowship training programs in Saudi Arabia: A nationwide cross-sectional study

**DOI:** 10.1016/j.amsu.2020.07.025

**Published:** 2020-07-23

**Authors:** Ameera Balhareth, Mohammed Abdulrazzaq AlDuhileb, Fozan A Aldulaijan, Mohammed Yousef Aldossary

**Affiliations:** a Department of General Surgery, King Fahad Specialist Hospital-Dammam, Dammam, Saudi Arabia

**Keywords:** COVID-19, Pandemic, Residency training, Fellowship training, Survey, Saudi Arabia

## Abstract

**Background:**

Coronavirus disease 2019 (COVID-19) has profoundly impacted residency and fellowship training and education. However, how and to what extent the daily involvement of trainees in clinical and surgical activities was compromised by the COVID-19 pandemic is currently unknown.

**Materials and methods:**

We conducted an electronic survey. An invitation was sent through the executive training administration of the Saudi Commission for Health Specialties (SCFHS) randomly to 400 residents and fellows over two weeks period from April 23, 2020 until May 6, 2020. Descriptive statistics were presented using counts and proportions (%). The comparison between the trainees among the socio-demographic and the characteristics of trainees toward the impact of COVID-19 pandemic on their training had been conducted using the Chi-square test. A p-value cut off point of 0.05 at 95% Confidence Interval (CI) used to determine statistical significance.

**Results:**

Out of the 400 questionnaires distributed, 240 trainees responded, resulting in a response rate of 60%. The most frequently cited specialty was surgical (41.3%) and medical (38.3%). Approximately 43% of them had direct contact with patients with COVID-19, and 43.8% had enough training regarding the proper use of Personal Protective Equipment (PPE). There were seven responders (2.9%) who had been infected by the disease. Among them, 6 (2.5%) members of their family had also been infected. Approximately 84.6% reported a reduction in training activities due to the current pandemic. Of those with surgical specialties, almost all (97%) reported that their surgical exposure reduced due to the COVID-19 pandemic.

**Conclusion:**

The adoption of smart learning is critical. For those who have been affected by examination delays, we recommend continuing to revise steadily using webinars, podcasts, prerecorded sessions, and social media. Routine activities such as journal clubs and departmental teaching should continue through webinars, if possible.

## Highlights

• The first cross-sectional survey study to show the impact of COVID-19 pandemic on training programs in Saudi Arabia.
• Almost 97% of the participants reported that their surgical exposure was reduced due to the pandemic.
• We recommend continuing to revise steadily using webinars, podcasts, prerecorded sessions, and social media.
• Routine activities such as journal clubs and departmental teaching should continue through webinars if possible.

## 1 Introduction

On December 31, 2019, the Chinese authorities notified the World Health Organization (WHO) regarding a novel coronavirus that has spread in Wuhan city as a highly contagious disease that affects the respiratory system [1]. The WHO declared the COVID-19 outbreak a pandemic on March 11, 2020 [2]. As for May 27, 2020, Saudi Arabia reported more than 76,000 patients and announced 411 deaths. In this unprecedented scenario, healthcare systems had to rapidly reshape their organization to cope with the emergency, aiming to optimize resources and minimize a further spread of the infection. Notably, addressing this challenge caused an inevitable shift from patient-centered medicine to a community-centered approach [1]. As such, hospitals all over the kingdom faced the challenge of reviewing their prioritization strategies regarding out-patient, in-patient services, and surgical procedures [3]. Besides, many non-specialized physicians and surgeons across the country had to dedicate part, if not all, of their practice to manage COVID-19 patients. Overall, this process has rapidly led to a substantial decrease in clinical and surgical practice across all hospitals in Saudi Arabia. In this context, the residents and fellows training might be critically affected [4]. However, how and to what extent the daily involvement of trainee residents and fellows in clinical and surgical activities was compromised by the COVID-19 pandemic is currently unknown. Although there has been a developing literature base defining the early clinical course of COVID-19 and aspects of critical care correlated to treating these patients, there has been a lack of evidence on how this pandemic will affect the educational programs, especially surgical training. Based on an online survey among our trainees in Saudi Arabia, which was performed in the period between April and May 2020, we publish these data demonstrating the significant impact of this pandemic on both educational and psychological aspects and explore possible solutions.

## 2 Materials and Methods

### 2.1 The questionnaire

The questionnaire had 34 questions and designed using SurveyMonkey® to capture the impact of COVID-19 pandemic on training quality of the residents and fellows enrolled in the SCFHS training programs in Saudi Arabia. The questions are designed along with two thematic blocks. The first one looks into the demographic data of the trainees involved in the questionnaire and are related to their personal life and training details. The second focuses on the impact of the COVID-19 pandemic on the quality of training and the psychological effects on the trainees. The questionnaire combined yes-no questions and multiple-choice questions with predefined answers offering respondents the possibility to choose and rank among several options or the possibility to grade on a “Never” to “Always” scale. The work has been reported in line with the STROCSS criteria [5].

### 2.2 Inclusion criteria

All medical/surgical residents and fellows under the training of SCFHC training programs in Saudi Arabia. None SCFHS training programs, medical interns, general practitioners, registrars, senior registrars, and consultants were all excluded from the study.

### 2.3 Survey sample

This study was conducted at King Fahad Specialist Hospital-Dammam as a primary site after obtaining institutional board review approval from our hospital ethical review committee. It was issued a (reference number SUR0431). This study registered with Research Registry (unique identifying number of research registry 5701). We conducted an electronic survey. An invitation was sent through the executive training administration of SCFHS randomly to 400 residents and fellows who are enrolled in one of the training programs of the SCFHS in 5 regions of Saudi Arabia (Northern, Eastern, Western, Middle, Southern) over two weeks period from April 23, 2020 until May 6, 2020. They received an invitation to participate in using official emails. A second reminder distributed after one week. At the end of the collecting period, 240 out of 400 respondents who completed and submitted the questionnaire. The survey response rate was 60%.

### 2.4 Questionnaire validation

The demographic items were on nominal and ordinal levels of measurement, and since they.

Represented personal information of the respondents, it was not necessary to assess their.

Validity. In contrast, the variables on challenges faced by respondents were meant to.

Supplement the understanding of the factors that may influence the residency and fellowships-training programs with COVID-19 pandemic. Consequently, along with an inconsistent scale involving nominal, ordinal, and interval variables, it was not crucial to validate the items statistically. However, the variables for the impact of COVID-19 pandemic on residency and fellowships-training programs formed the core part of the research questionnaire. They were measured using a 5-point Likert scale, and it was important to validate them to make sure that they were accurate in measuring what they purported to measure. The results of the validity test using the Pearson correlation (ρ) method established that all the five items were valid at 95% confidence level.

### 2.5 Research reliability

The research conducted a reliability test to determine whether the five items used to measure the impact of COVID-19 pandemic on residency and fellowships-training programs were consistent and would produce the same results if they were to be used more than one time. The criteria used for this determination was the Cronbach's alpha, in which an item is considered reliable if the obtained value is greater than 0.6. The results showed that the questions measurement was reliable (5 items; α = 0.790).

### 2.6 Statistical analysis

Descriptive statistics were presented using counts and proportions (%). The comparison between the resident's levels and fellows among the socio-demographic and the characteristics of residents and fellows toward the impact of COVID-19 pandemic on their training had been conducted using the Chi-square test. A p-value cut off point of 0.05 at 95% CI used to determine statistical significance. All statistical data were analyzed using the Statistical Package for Social Sciences, version 21 (SPSS, Chicago, IL, USA).

## 3 Results

We recruited 240 residents and fellows to measure the impact of COVID-19 pandemic on their training. Table 1 presented the basic demographic data of the residents and fellows. Approximately 60% of residents were females, with 26–30 years old was the most common age group (72.9%). 57.9% of the residents and fellows were unmarried, with most of them have no children (73.3%). The most frequently cited specialty was surgical (41.3%) and medical (38.3%). When comparing the resident levels and fellows among the basic demographic data of residents and fellows, it found that age group (P < 0.001), marital status (P = 0.048), and having children (P = 0.001) had significantly influence toward resident levels and fellows.

**Table 1:** Socio-demographic characteristics according to residents levels and fellows.

Study variables	Overall N (%) ^(n=240)^	Junior resident N (%) ^(n=136)^	Senior resident N (%) ^(n=77)^	Fellow N (%) ^(n=27)^	P-value§
Gender					
• Male	97 (40.4%)	50 (36.8%)	33 (42.9%)	14 (51.9%)	0.300
• Female	143 (59.6%)	86 (63.2%)	44 (57.1%)	13 (48.1%)	
Age group					
• 21–25 years	27 (11.3%)	23 (16.9%)	0	04 (14.8%)	< **0.001**∗∗
• 26–30 years	175 (72.9%)	105 (77.2%)	64 (83.1%)	06 (22.2%)	
• >30 years	38 (15.8%)	08 (05.9%)	13 (16.9%)	17 (63.0%)	
Marital Status					
• Unmarried	139 (57.9%)	88 (64.7%)	37 (48.1%)	14 (51.9%)	**0.048**∗∗
• Married	101 (42.1%)	48 (35.3%)	40 (51.9%)	13 (48.1%)	
Having children					
• Yes	64 (26.7%)	24 (17.6%)	30 (39.0%)	10 (37.0%)	**0.001**∗∗
• No	176 (73.3%)	112 (82.4%)	47 (61.0%)	17 (63.0%)	
Specialty					
• Medical	92 (38.3%)	56 (41.2%)	28 (36.4%)	08 (29.6%)	0.104
• Surgical	99 (41.3%)	62 (45.6%)	25 (32.5%)	12 (44.4%)	
• ICU or Anesthesia	07 (02.9%)	05 (03.7%)	02 (02.6%)	0	
• Emergency medicine	08 (03.3%)	02 (01.5%)	04 (05.2%)	02 (07.4%)	
• Radiology	12 (05.0%)	05 (03.7%)	06 (07.8%)	01 (03.7%)	
• Pathology	03 (01.3%)	01 (0.70%)	02 (02.6%)	0	
• Others	19 (07.9%)	05 (03.7%)	10 (13.0%)	04 (14.8%)	

§ P-value has been calculated using Chi-square test.

∗∗ Significant at p < 0.05 level.

Table 2 described the impact of COVID-19 pandemic with regard to stress and support. Based on the results, more than three quarters (76.3%) of the residents and fellows worked in the quarantine area. When asked if they were obligated to change hospital due to pandemic, a little below half of them (46.7%) were forced into it. Furthermore, approximately 43% of them had direct contact with the patients with COVID-19, and 43.8% had enough training regarding the proper use of PPE. When rating the availability of PPE in their hospital, more than half of them (52.9%) rated “most of the time.” When asked the rating if their program director and institute had their full support, approximately 39% rated “most of the time” or 31.7% rated “always.” Likewise, 71.7% reported that they got virtual teaching on their continuing education, while more than half (53.8%) understood their role in the current situation. When asked about their rating, whether they are anxious or worried about the situation, 45% of them rated “most of the time.” When asked about their rating, if they feel a low mood, 37.1% reported “most of the time.” When asked about their rating on feeling alone due to the current situation, about one quarter (32.5%) said “most of the time.” When asked if they aware of the new management protocols due to the current COVID-19 pandemic, nearly half of them (47.1%) rated “most of the time.” Regarding the impact of COVID-19 pandemic in stress and support to the level of residency and fellowship, we have learned that statements such as “Were you obliged to change the hospital because of the pandemic?” (P = 0.037) and “Do you feel low mood?” (P = 0.047) had a significant impact on the residency levels and fellows.

**Table 2:** Impact of COVID-19 pandemic in stress and support in accordance to residency levels and fellows.

Statement	Overall N (%) ^(n=240)^	Junior resident N (%) ^(n=136)^	Senior residentN (%) ^(n=77)^	Fellow N (%) ^(n=27)^	P-value §
Do you work in quarantine area					
• Yes	183 (76.3%)	104 (76.5%)	59 (76.6%)	20 (74.1%)	0.961
• No	57 (23.8%)	32 (23.5%)	18 (23.4%)	07 (25.9%)	
Were you obliged to change the hospital because of this pandemic?					
• Yes	112 (46.7%)	63 (46.3%)	42 (54.5%)	07 (25.9%)	**0.037**∗∗
• No	128 (53.3%)	73 (53.7%)	35 (45.5%)	20 (74.1%)	
Do you get direct contact with COVID-19 patient?					
• Yes	103 (42.9%)	57 (41.9%)	39 (50.6%)	07 (25.9%)	0.077
• No	137 (57.1%)	79 (58.1%)	38 (49.4%)	20 (74.1%)	
Did you get training on site for PPE in advance?					
• Yes	105 (43.8%)	54 (39.7%)	35 (45.5%)	16 (59.3%)	0.163
• No	135 (56.3%)	82 (60.3%)	42 (54.5%)	11 (40.7%)	
Do you have enough PPE available in the hospital?					
• Always	70 (29.2%)	38 (27.9%)	22 (28.6%)	10 (37.0%)	0.115
• Most of the time	127 (52.9%)	78 (57.4%)	41 (53.2%)	08 (29.6%)	
• Rarely	34 (14.2%)	16 (11.8%)	12 (15.6%)	06 (22.2%)	
• Never	09 (03.8%)	04 (02.9%)	02 (02.6%)	03 (11.1%)	
Do you get full support from your program director and institute?					
• Always	76 (31.7%)	49 (36.0%)	16 (20.8%)	11 (40.7%)	0.312
• Most of the time	93 (38.8%)	50 (36.8%)	33 (42.9%)	10 (37.0%)	
• Rarely	46 (19.2%)	23 (16.9%)	19 (24.7%)	04 (14.8%)	
• Never	25 (10.4%)	14 (10.3%)	09 (11.7%)	02 (07.4%)	
Do you get any form of virtual teaching?					
• Yes	172 (71.7%)	99 (72.8%)	53 (68.8%)	20 (74.1%)	0.792
• No	68 (28.3%)	37 (27.2%)	24 (31.2%)	07 (25.9%)	
Do you understand your role in this situation					
• Yes	129 (53.8%)	69 (50.7%)	44 (57.1%)	16 (59.3%)	0.461
• No	28 (11.7%)	16 (11.8%)	07 (09.1%)	05 (18.5%)	
• Not sure	83 (34.6%)	51 (37.5%)	26 (33.8%)	06 (22.2%)	
Do you feel anxious and worried about the situation?					
• Always	65 (27.1%)	33 (24.3%)	26 (33.8%)	06 (22.2%)	0.441
• Most of the time	108 (45.0%)	65 (47.8%)	30 (39.0%)	13 (48.1%)	
• Rarely	54 (22.5%)	33 (24.3%)	16 (20.8%)	05 (18.5%)	
• Never	13 (05.4%)	05 (03.7%)	05 (06.5%)	03 (11.1%)	
Do you feel low mood?					
• Always	66 (27.5%)	34 (25.0%)	28 (36.4%)	04 (14.8%)	**0.047**∗∗
• Most of the time	89 (37.1%)	54 (39.7%)	24 (31.2%)	11 (40.7%)	
• Rarely	70 (29.2%)	41 (30.1%)	22 (28.6%)	07 (25.9%)	
• Never	15 (06.3%)	07 (05.1%)	03 (03.9%)	05 (18.5%)	

Statement	Overall N (%) ^(n=240)^	Junior resident N (%) ^(n=136)^	Senior resident N (%) ^(n=77)^	Fellow N (%) ^(n=27)^	P-value^§^
Do you feel you are lonely in this time?					
• Always	53 (22.1%)	28 (20.6%)	20 (26.0%)	05 (18.5%)	0.738
• Most of the time	78 (32.5%)	47 (34.6%)	25 (32.5%)	06 (22.2%)	
• Rarely	64 (26.7%)	35 (25.7%)	20 (26.0%)	09 (33.3%)	
• Never	45 (18.8%)	26 (19.1%)	12 (15.6%)	07 (25.9%)	
Are you aware of the new management protocols that are related to your specialty which have been generated in COVID-19 pandemic?					
• Always	50 (20.8%)	24 (17.6%)	17 (22.1%)	09 (33.3%)	0.133
• Most of the time	113 (47.1%)	59 (43.4%)	43 (55.8%)	11 (40.7%)	
• Rarely	58 (24.2%)	39 (28.7%)	13 (16.9%)	06 (22.2%)	
• Never	19 (07.9%)	14 (10.3%)	04 (05.2%)	01 (03.7%)	

§ P-value has been calculated using Chi-square test.

∗∗ Significant at p < 0.05 level.

In Table 3, we reported the impact of COVID-19 pandemic on self and family wellbeing. As revealed, 7 residents and fellows (2.9%) infected by the disease. Among them, 6 (2.5%) members of their family were infected, although only one resident reported that it was because of him/her. When asked about their rating, if they feel safe and protected, more than half (53.8%) rated “most of the time.”

**Table 3:** Impact of COVID-19 pandemic to Self and family welling in accordance to residency levels and fellows.

Statement	Overall N (%) ^(n=240)^	Junior resident N (%) ^(n=136)^	Senior resident N (%) ^(n=77)^	Fellow N (%) ^(n=27)^	P-value§
Did you get infected as results of working exposure?					
• Yes	07 (02.9%)	02 (01.5%)	03 (03.9%)	02 (07.4%)	0.203
• No	233 (97.1%)	134 (98.5%)	74 (96.1%)	25 (92.6%)	
Did any member of your family get infected?					
• Yes	06 (02.5%)	02 (01.5%)	02 (02.6%)	02 (07.4%)	0.196
• No	234 (97.5%)	134 (98.5%)	75 (97.4%)	25 (92.6%)	
If the answer was yes, is it because of you directly? ∗					
• Yes	01 (16.7%)	0	01 (50.0%)	0	0.301
• No	05 (83.3%)	02 (100%)	01 (50.0%)	02 (100%)	
Do you feel safe and protected?					
• Always	09 (03.8%)	03 (02.2%)	02 (02.6%)	04 (14.8%)	**0.016**∗∗
• Most of the time	129 (53.8%)	68 (50.0%)	44 (57.1%)	17 (63.0%)	
• Rarely	81 (33.8%)	50 (36.8%)	25 (32.5%)	06 (22.2%)	
• Never	21 (08.8%)	15 (11.0%)	06 (07.8%)	0	
Do you feel that your family is safe?					
• Always	14 (05.8%)	08 (05.9%)	03 (03.9%)	03 (11.1%)	0.679
• Most of the time	122 (50.8%)	67 (49.3%)	39 (50.6%)	16 (59.3%)	
• Rarely	69 (28.8%)	39 (28.7%)	24 (31.2%)	06 (22.2%)	
• Never	35 (14.6%)	22 (16.2%)	11 (14.3%)	02 (07.4%)	
Are you maintaining good life style? †					
• Health	122 (50.8%)	63 (46.3%)	44 (57.1%)	15 (55.6%)	0.276
• Food	121 (42.1%)	64 (47.1%)	42 (54.5%)	15 (55.6%)	0.491
• Sleep	101 (42.1%)	61 (44.9%)	30 (39.0%)	10 (37.0%)	0.601
• Exercise	57 (23.8%)	30 (22.1%)	17 (22.1%)	10 (37.0%)	0.227
Are you away from family?					
• Yes	103 (42.9%)	57 (41.9%)	33 (42.9%)	13 (48.1%)	0.836
• No	137 (57.1%)	79 (58.1%)	44 (57.1%)	14 (51.9%)	
Did you change your residence to protect your family?					
• Yes	44 (18.3%)	24 (17.6%)	15 (19.5%)	05 (18.5%)	0.946
• No	196 (81.7%)	112 (82.4%)	62 (80.5%)	22 (81.5%)	

∗ Only those residents with infected family members were included in the analysis.

† Variable with multiple responses.

§ P-value has been calculated using Chi-square test.

∗∗ Significant at p < 0.05 level.

Concerning the safety of their family, the result was almost identical, with 50.8% rated “most of the time.” The proportion of residents and fellows who maintained a good lifestyle in health, food, sleep, and exercise was 50.8%, 42.1%, 42.1%, and 23.8%, respectively. Besides, approximately 43% of the residents and fellows were working away from their families. Few of them (18.3%) had changed their residence location to protect their family. When measuring the impact of COVID-19 pandemic to self and family wellbeing according to the level of residency and fellows, it was found that being safe and protected significantly influences the level of residency and fellows (P = 0.016).

Table 4 showed the impact of COVID-19 pandemic in resident and fellow examination and SCFHS. Based on our assessment, the proportion of residents and fellows who missed the exam during the pandemic was only 10%, while nearly all (84.6%) reported a reduction in the training activities due to the current pandemic. Of those with surgical specialties, almost all (97%) reported that their surgical exposure reduced as a result of the COVID-19 pandemic. When asked about their rating, whether they have enough time to read and study during the pandemic and that whether they were psychologically prepared to do it, 41.3% and 43.3% rated “most of the time” and “rarely,” respectively. When asked if they feel stress because of the upcoming exams during the pandemic, more than half of them (57.9%) rated “always.” When measuring the impact of the pandemic in examination and SCFHS among residency levels and fellows, it found that statement about “Psychologically prepared to study and read” (P < 0.001) and “Feeling stress because of the upcoming exams” (P < 0.001) had a significant impact to the level of residency and fellows.

**Table 4:** Impact of COVID-19 pandemic in Examination and SCFHS in accordance to residency levels and Fellows.

Statement	Overall N (%) ^(n=240)^	Junior residentN (%) ^(n=136)^	Senior resident N (%) ^(n=77)^	Fellow N (%) ^(n=27)^	P-value§
Did you miss an exam during the pandemic?					
• Yes	24 (10.0%)	14 (10.3%)	07 (09.1%)	03 (11.1%)	0.941
• No	216 (90.0%)	122 (89.7%)	70 (90.9%)	24 (88.9%)	
Is there a reduction in the training activities during COVID-19 pandemic?					
• Yes	203 (84.6%)	117 (86.0%)	63 (81.8%)	23 (85.2%)	0.713
• No	37 (15.4%)	19 (14.0%)	14 (18.2%)	04 (14.8%)	
For surgical specialties, is there a reduction in the level of surgical exposure and the number of operations during COVID-19 pandemic? ∗					
• Yes	96 (97.0%)	61 (98.4%)	23 (92.0%)	12 (100%)	0.235
• No	03 (01.3%)	01 (01.6%)	02 (08.0%)	0	
Do you have enough time to read and study during the pandemic?					
• Always	36 (15.0%)	16 (11.8%)	11 (14.3%)	09 (33.3%)	0.068
• Most of the time	99 (41.3%)	61 (44.9%)	27 (35.1%)	11 (40.7%)	
• Rarely	75 (31.3%)	44 (32.4%)	26 (33.8%)	05 (18.5%)	
• Never	30 (12.5%)	15 (11.0%)	13 (16.9%)	02 (07.4%)	
Do you feel that you are psychologically prepared to study and read?					
• Always	14 (05.8%)	05 (03.7%)	03 (03.9%)	06 (22.2%)	< **0.001**∗∗
• Most of the time	43 (17.9%)	30 (22.1%)	08 (10.4%)	05 (18.5%)	
• Rarely	104 (43.3%)	59 (43.4%)	30 (39.0%)	15 (55.6%)	
• Never	79 (32.9%)	42 (30.9%)	36 (46.8%)	01 (03.7%)	
Do you feel stress because of the upcoming exams during the pandemic?					
• Always	139 (57.9%)	80 (58.8%)	52 (67.5%)	07 (25.9%0	< **0.001**∗∗
• Most of the time	69 (28.8%)	41 (30.1%)	19 (24.7%)	09 (33.3%)	
• Rarely	23 (09.6%)	10 (07.4%)	06 (07.8%)	07 (25.9%)	
• Never	09 (03.8%)	05 (03.7%)	0	04 (14.8%)	

∗ Only those residents with surgical specialties were included in the analysis.

§ P-value has been calculated using Chi-square test.

∗∗ Significant at p < 0.05 level.

## 4 Discussion

One of the most immediate changes introduced to the training programs has been the broad canceling of in-person medical meetings and conferences, mostly being replaced by recorded lectures, live-streams, or webinars. One reason is to flatten the curve, to minimize personal interactions to mitigate and contain the spread of COVID-19. Another reason is to decrease the risk of exposure for trainees, which is an understandable concern. However, many are willing to put themselves at risk and, as such, can be frustrated by these decisions. Moreover, with the current lack of personal protective equipment, canceling clerkships is necessary to ensure that healthcare workers are adequately able to protect themselves during this pandemic. Daily virtual learning has become the primary form of collaboration between residents/fellows and tutors; increased use of telematics educational programs (as telemedicine and telementoring of surgical procedures) could be the opportunity to bridge the training gap. Crises like this are opportunities for medical educators to leverage technology for both undergraduate and postgraduate medical education. While newer initiatives such as webcasts are increasingly being adopted, in-person didactic lectures and tutorials remain a significant cornerstone of medical education. Given the highly infectious nature of COVID-19, and likewise, most emerging infections, face-to-face interactions in large-group settings (such as lectures) can potentially be hotbeds for disease spread and transmission. The American College of Surgeons, the Centers for Medicare and Medicaid Services, and the United States Surgeon General have recommended a delay of all elective and non-essential medical and surgical procedures to minimize the spread of disease and conserve medical supplies and personal protective equipment to avoid crisis levels needed for sick patients [6]. New guidelines from the American Board of Medical Specialties and Accreditation Council for Graduate Medical Education have emerged and continue to evolve to achieve milestones and modify training log requirements while still able to satisfy all graduation competencies. Many residency programs have created teams where a group of the residents is assigned to clinical activities and duties, while others are to abstain as standby teams [7]. The non-clinical educational activities were conducted using video conferencing and virtual meetings with their mentor [8]. The involvement of the trainees in on-call duty was not particularly reduced, outlining the essential nature of this activity, especially in high-volume centers. The significant decrease in trainees' participation in outpatient clinics can be explained by their forced cessation of the clinics to minimize the human contact of the non-urgent cases [8]. To keep the residents and fellows exposed to this activity, strategies taking advantage of telemedicine and virtual clinics should be implemented [8]. The reduction of trainees’ involvement in diagnostic activities may be partly due to deferring elective, non-urgent procedures and operations, partly to the higher proportion of consultants performing such activities during the emergency, aiming to minimize the number of healthcare workers exposed to hospital-acquired infections [9].

Similarly, the decrease in trainees' exposure to all operational procedures can be explained, considering the recent recommendations to limit surgical procedures to experienced surgeons [10]. This is even more relevant for minimally invasive surgery, due to the increasing concerns regarding its safety in light of the potential risk of dissemination of COVID-19 infection via laparoscopic gas [9]. Moreover, the suspension of all deferrable surgeries, with the consequent reduction of daily operational procedures, further contributes to explain this finding [11]. To outwit this, technology, such as, video-conferencing [12] and e-learning platforms [13,14], can be used to deliver lectures or tutorials remotely via smartphones, tablets, and laptops. Faculty, residents, and fellows can then log in at designated times for discussions, which can be facilitated in real-time via teleconferencing applications. In addition to lectures, teleconferencing can also be used to demonstrate medical procedures and surgical techniques [12]. The online theoretical courses and departmental simulators are vital resources to keep surgical skills going and prepare for the examination. Video-based surgical demonstrations are available as an adjunct to the live surgeries. With the new era of technology and telemedicine, the effects of COVID-19 pandemic on residents and fellows training can hopefully be kept to a minimum. There are another challenges and an unfortunately largely ignored or missed aspect of such crises, which is the impact on the mental health of both health care workers and patients. As shown in our survey, trainees in Saudi Arabia had their share of this impact in various degrees, leading to mixed feelings and emotional disturbance. Mental illness, behavioral change, and emotional distress are all known to be caused by disaster events [14,15]. Trainee residents and fellows who are based in hospitals and who are in direct contact with suspected or confirmed cases of COVID-19 are the most susceptible to get infected with the disease. This has a significant impact on their mental health for many reasons. Contracting the virus is one factor, but the fear of infecting others like families and friends is a major concern to them as well [16]. Depression, anxiety, and frustration in various degrees were reported among healthcare professionals in hospitals dealing with Severe Acute Respiratory Syndrome (SARS) outbreaks [17]. Medical staff working in first-line units like emergency departments, intensive care units, and infectious disease showed twice more likely to have depression and anxiety than other medical staff who work in departments that are less likely to come in close contact with infected patients [18]. The feeling of uncertainty exacerbated by modification of recommendations and infection control protocols is another cause of anxiety among healthcare workers [19]. Unfortunately, health professionals and mainly junior staff are not prepared with psychological and mental health care training to cope and deal with such crises [16,20]. So as health professionals’ mental health has been clearly proved to be affected by such health disasters, understanding it and its needs during such events might help medical professionals be prepared both mentally and physically to fight this war [21]. Health authorities should provide all kinds of support for health professionals during disease outbreaks. These interventions multidisciplinary mental healthcare teams that can include psychiatrists, social workers, and other mental health workers [16,17]. Healthcare workers and especially junior staff, should have regular updates on all aspects related to the outbreak with clear communication to deal with the perception of uncertainty and fear [22]. Social support in the form of support from health institutions, colleagues, family, and friends has been shown to greatly help and positively affect health care providers dealing with this such pandemics [23].

### 4.1 Limitations and future study

In completing the study, the researchers acknowledged several limitations, including the lack of time, financial resources, and limited the number of respondents (240). The use of such a small sample size to generalize the whole field could affect the feasibility of the findings. Consequently, using responses from only 240 trainees to generalize on the residency and fellowship training programs could have adverse effects. To improve the effectiveness of the study, a longitudinal study design should be used. Another future study needed to assess the trainees' satisfaction toward the SCFHS decisions in canceling/postponing exams to specific specialties.

## 5 Conclusion

There are obvious training impacts and work plan changes across all countries all over the world. The slowdown of residents' and fellows’ learning curve is inevitable, so the adoption of smart learning is critical. For those who have been affected by examination delays, we recommend continuing to revise steadily using webinars, podcasts, prerecorded sessions, and social media. Routine activities such as journal clubs and departmental teaching should continue through webinars, if possible. This crisis has a great impact on the mental health of both healthcare workers and patients. High levels of anxiety and depression were noticed, which emphasizing the need for psychological supporting programs for the trainees at all levels.

## Ethical Approval

Ethical approval was obtained from the Institutional Review Board (IRB) of the King Fahad Specialist Hospital-Dammam, Dammam, Saudi Arabia. The ethical approval was signed on 4th June 2020 and was issued an IRB Study Number – SUR0341

## Consent

Electronic written informed consent was obtained from the participants for publication of this study. A copy of the written consent is available for review by the Editor-in-Chief of this journal on request

## Author contribution

Study concept or design – AB, MAD, FAA.

Data collection – AB, MAD, FAA.

Data interpretation – AB, MAD, FAA, MYD.

Literature review – AB, MAD, FAA, MYD.

Data analysis – AB.

Drafting of the paper – AB, MAD, FAA, MYD.

Editing of the paper – AB, MAD, FAA, MYD

## Registration of Research Studies

researchregistry5701

## Guarantor

Dr. Ameera Balhareth

## Funding sources

This study did not receive any funding from public or private sectors.

## Declaration of competing interest

The authors declare no competing interests.
