# Supplementary material for: Impact of COVID-19 pandemic on residency and fellowship training programs in Saudi Arabia: A nationwide cross-sectional study
Source: Ann Med Surg (Lond). 2020 Jul 23;57:127–32. doi: 10.1016/j.amsu.2020.07.025 (PMC7377677; doi:10.1016/j.amsu.2020.07.025)
Supplement: Multimedia component 2 [file mmc2.pdf]

Q1 Gender

Answered: 239 Skipped: 1

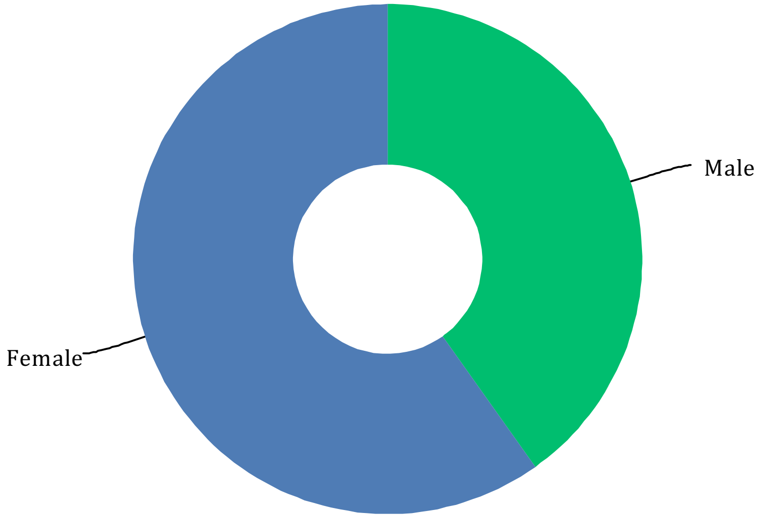

ANSWER CHOICES	RESPONSES	
Male	40.17%	96
Female	59.83%	143
TOTAL		239

Q2 Age

Answered: 240 Skipped: 0

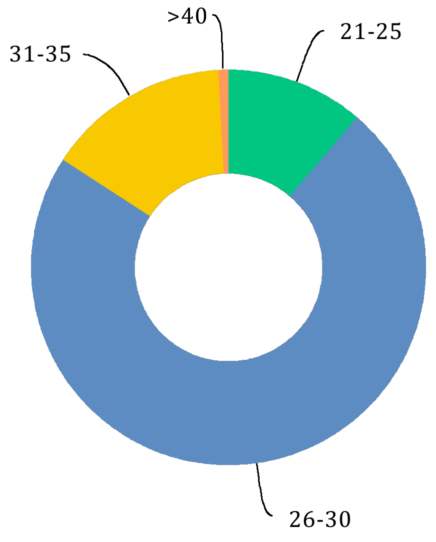

ANSWER CHOICES	RESPONSES	
21-25	11.25%	27
26-30	72.92%	175
31-35	15.00%	36
36-40	0.00%	0
>40	0.83%	2
TOTAL		240

Q3 Marital status

Answered: 240 Skipped: 0

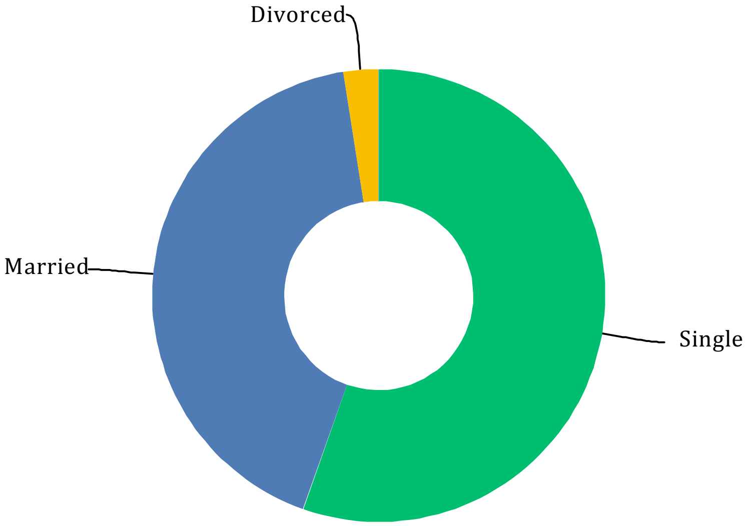

ANSWER CHOICES	RESPONSES	
Single	55.42%	133
Married	42.08%	101
Divorced	2.50%	6
Widow	0.00%	0
TOTAL		240

Q4 Number of children

Answered: 240 Skipped: 0

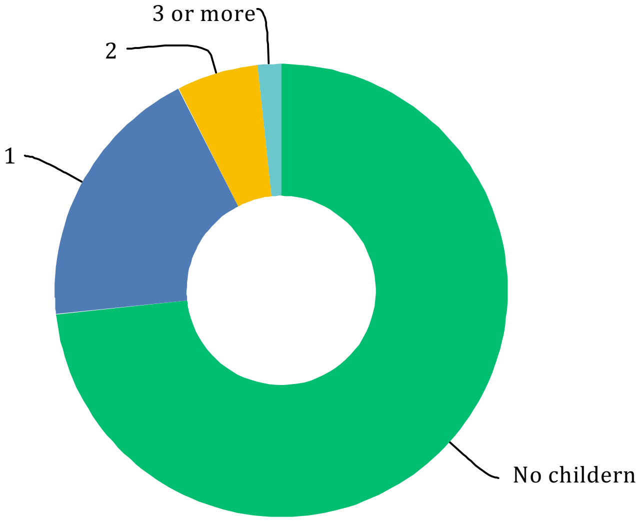

ANSWER CHOICES	RESPONSES	
No childern	73.33%	176
1	19.17%	46
2	5.83%	14
3 or more	1.67%	4
TOTAL		240

Q5 Level of training:

Answered: 240   Skipped: 0

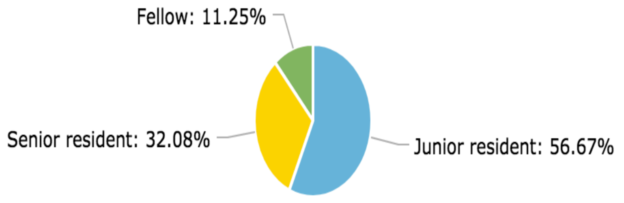

ANSWER CHOICES	RESPONSES	
Junior resident	56.67%	136
Senior resident	32.08%	77
Fellow	11.25%	27
TOTAL		240

Q6 Specialty

Answered: 240 Skipped: 0

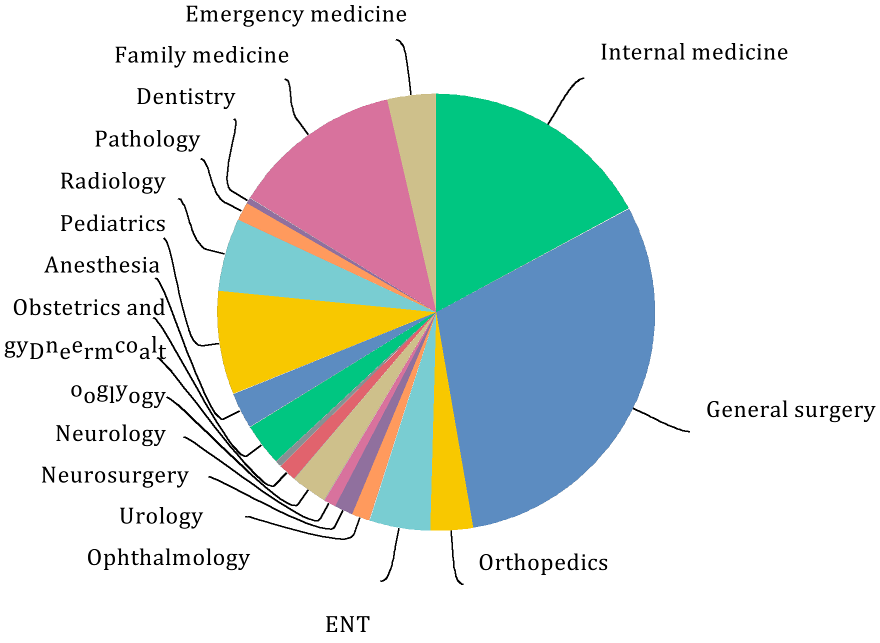

ANSWER CHOICES	RESPONSES	
Internal medicine (1)	15.83%	38
General surgery (2)	27.92%	67
Orthopedics (3)	2.92%	7
ENT (4)	4.17%	10
Ophthalmology (5)	1.25%	3
Urology (6)	1.25%	3
Neurosurgery (7)	0.83%	2
Neurology (8)	2.50%	6
Dermatology (9)	1.25%	3
Intensive care (10)	0.42%	1
Obstetrics and gynecology (11)	2.92%	7
Anesthesia (12)	2.50%	6
Pediatrics (13)	7.08%	17
Radiology (14)	5.00%	12
Pathology (15)	1.25%	3
Dentistry (16)	0.42%	1
Family medicine (17)	11.67%	28
Emergency medicine (18)	3.33%	8
TOTAL		240

BASIC STATISTICS

Minimum	Maximum	Median	Mean	Standard Deviation
1.00	18.00	3.00	7.14	6.29

Q7 Do you work in Quarantine area?

Answered: 240 Skipped: 0

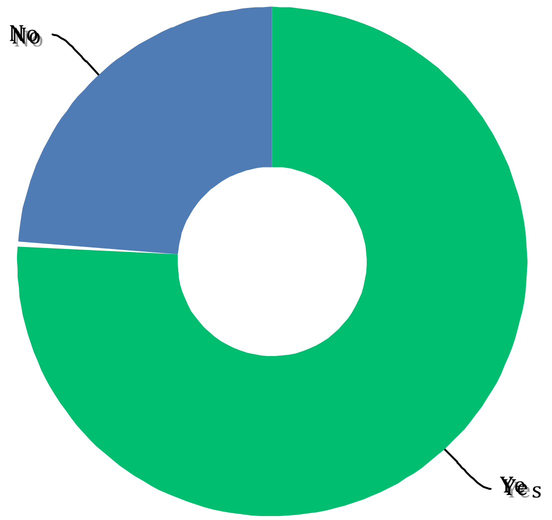

ANSWER CHOICES	RESPONSES	
Yes	76.25%	183
No	23.75%	57
TOTAL		240

Q8 Were you obliged to change the hospital because of this pandemic?

Answered: 240 Skipped: 0

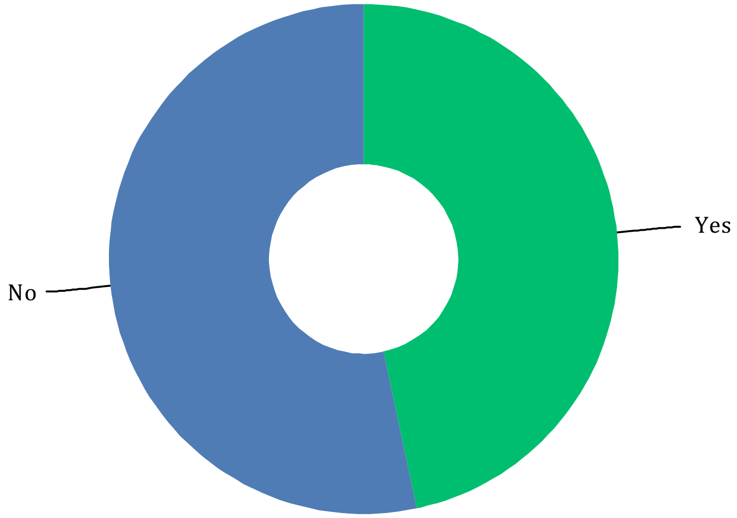

ANSWER CHOICES	RESPONSES	
Yes	46.67%	112
No	53.33%	128
TOTAL		240

Q9 Do you get direct contact with corona patient?

Answered: 240 Skipped: 0

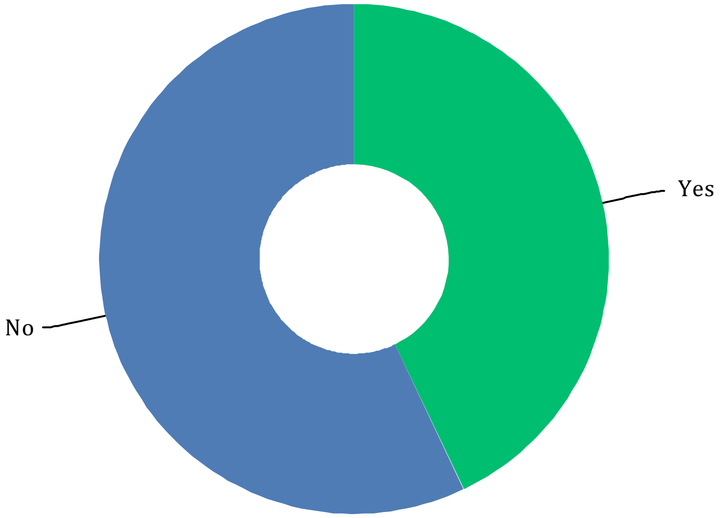

ANSWER CHOICES	RESPONSES	
Yes	42.92%	103
No	57.08%	137
TOTAL		240

Q10 Did you get training on site for PPE in advance?

Answered: 240 Skipped: 0

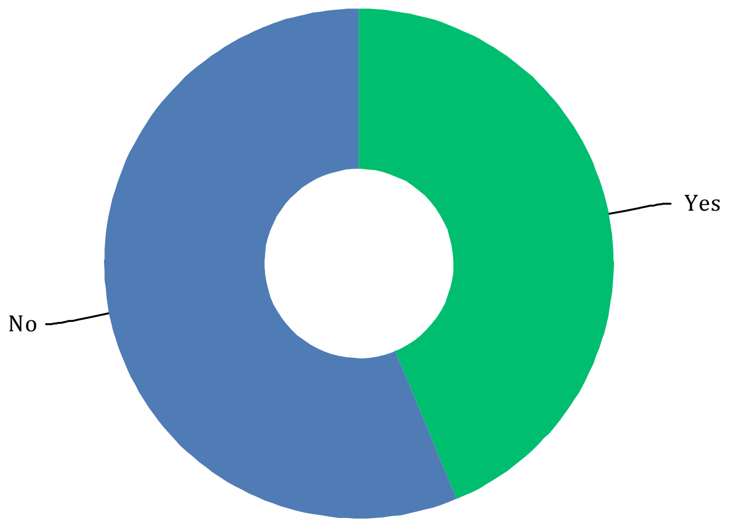

ANSWER CHOICES	RESPONSES	
Yes	43.75%	105
No	56.25%	135
TOTAL		240

Q11 Do you have enough PPE available in the hospital?

Answered: 240   Skipped: 0

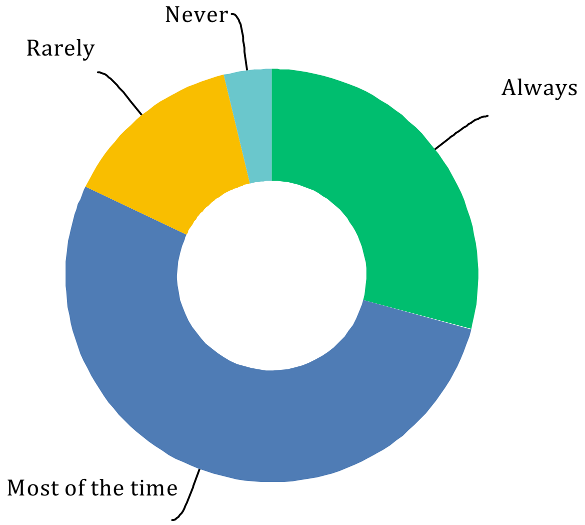

ANSWER CHOICES	RESPONSES	
Always	29.17%	70
Most of the time	52.92%	127
Rarely	14.17%	34
Never	3.75%	9
TOTAL		240

Q12 Do you get full support from your program director and institute?

Answered: 240   Skipped: 0

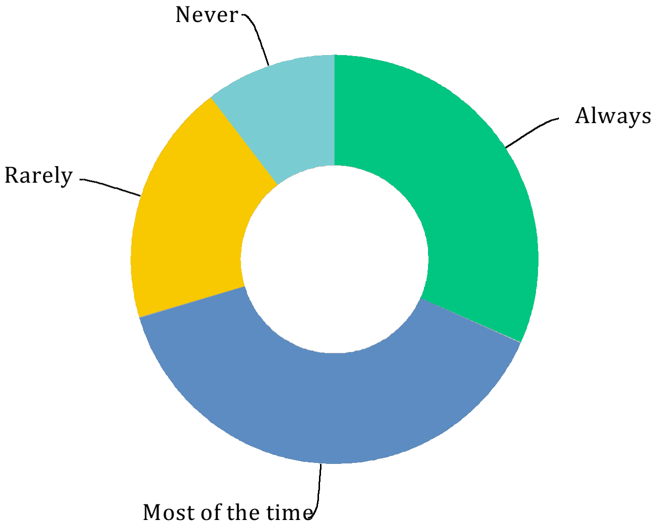

ANSWER CHOICES	RESPONSES	
Always	31.67%	76
Most of the time	38.75%	93
Rarely	19.17%	46
Never	10.42%	25
TOTAL		240

Q13 Do you get any form of virtual teaching?

Answered: 240   Skipped: 0

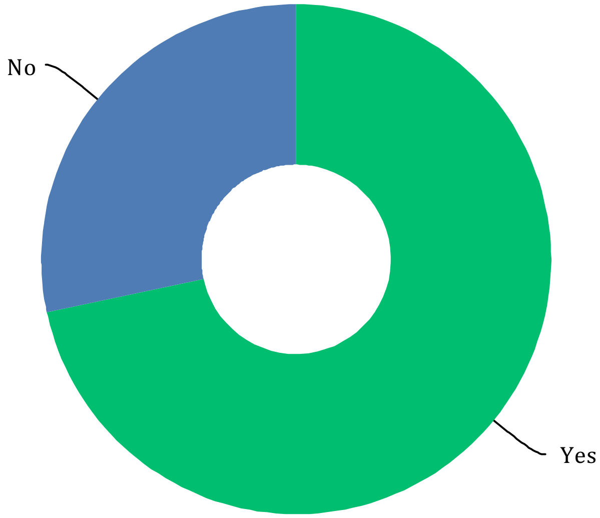

ANSWER CHOICES	RESPONSES	
Yes	71.67%	172
No	28.33%	68
TOTAL		240

Q14 Do you understand your role in this situation?

Answered: 240 Skipped: 0

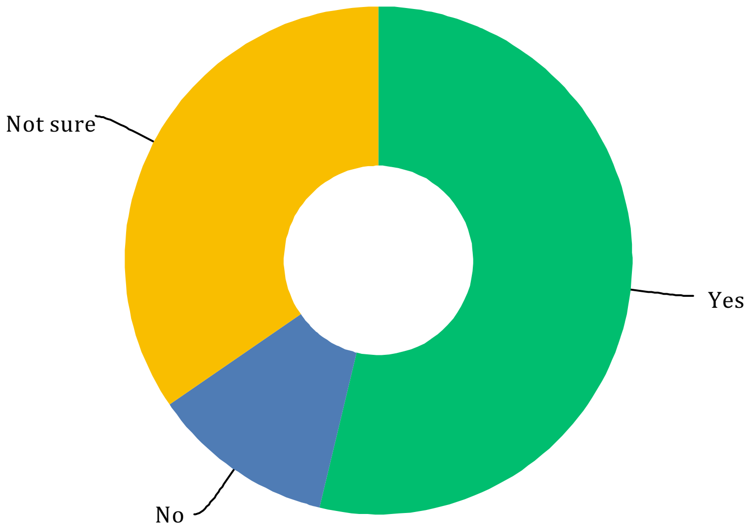

ANSWER CHOICES	RESPONSES	
Yes	53.75%	129
No	11.67%	28
Not sure	34.58%	83
TOTAL		240

Q15 Do you feel anxious and worried about the situation?

Answered: 240 Skipped: 0

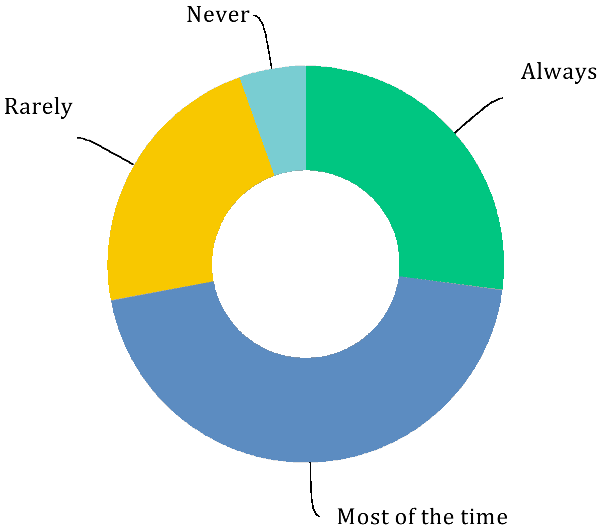

ANSWER CHOICES	RESPONSES	
Always	27.08%	65
Most of the time	45.00%	108
Rarely	22.50%	54
Never	5.42%	13
TOTAL		240

Q16 Do you feel low mood?

Answered: 239 Skipped: 1

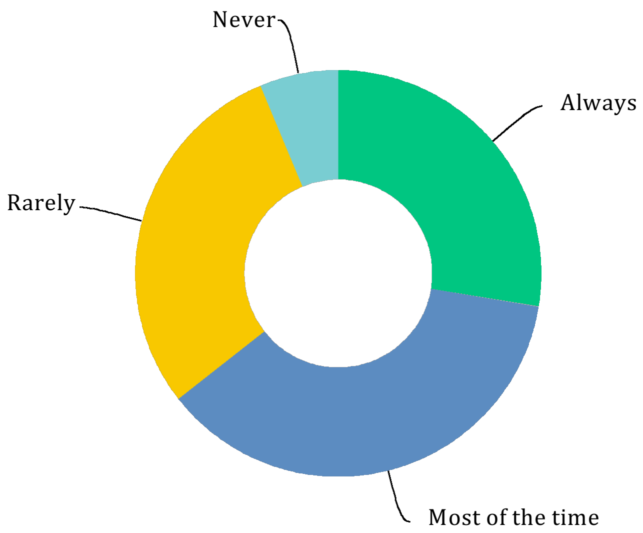

ANSWER CHOICES	RESPONSES	
Always	27.62%	66
Most of the time	36.82%	88
Rarely	29.29%	70
Never	6.28%	15
TOTAL		239

Q17 Do you feel you are lonely in this time?

Answered: 240 Skipped: 0

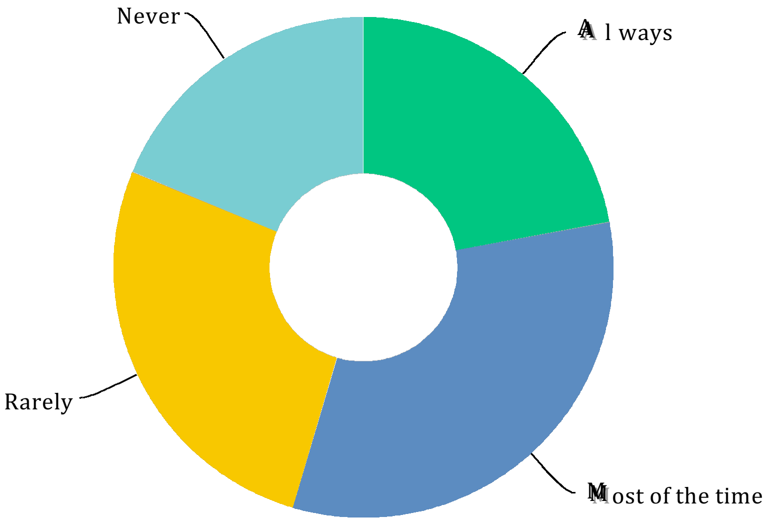

ANSWER CHOICES	RESPONSES	
Always	22.08%	53
Most of the time	32.50%	78
Rarely	26.67%	64
Never	18.75%	45
TOTAL		240

Q18 Are you aware of the new management protocols that are related to your specialty which have been generated in COVID-19 pandemic?

Answered: 239 Skipped: 1

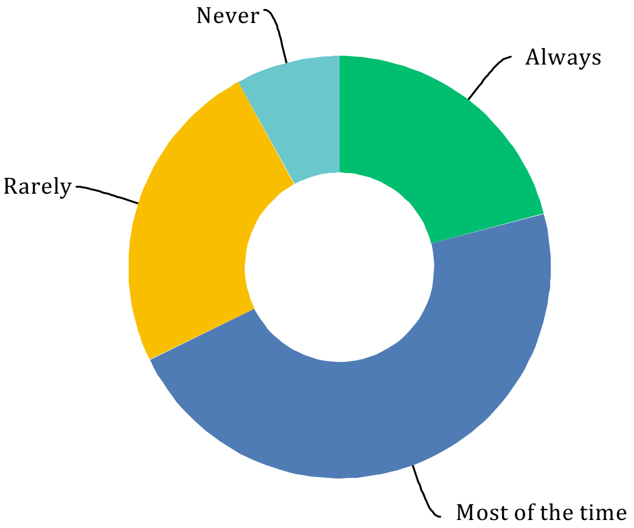

ANSWER CHOICES	RESPONSES	
Always	20.92%	50
Most of the time	46.86%	112
Rarely	24.27%	58
Never	7.95%	19
TOTAL		239

Q19 Did you get infected as results of working exposure?

Answered: 239 Skipped: 1

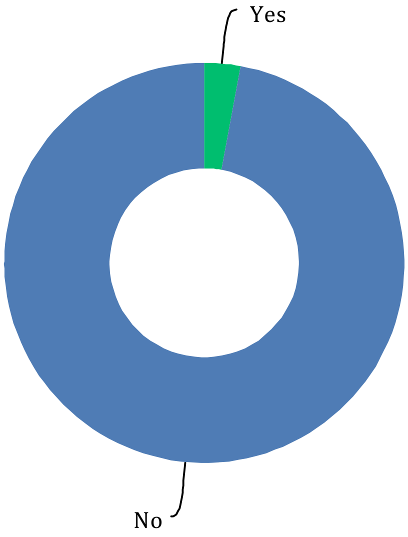

ANSWER CHOICES	RESPONSES	
Yes	2.93%	7
No	97.07%	232
TOTAL		239

Q20 Did any member of your family get infected?

Answered: 239 Skipped: 1

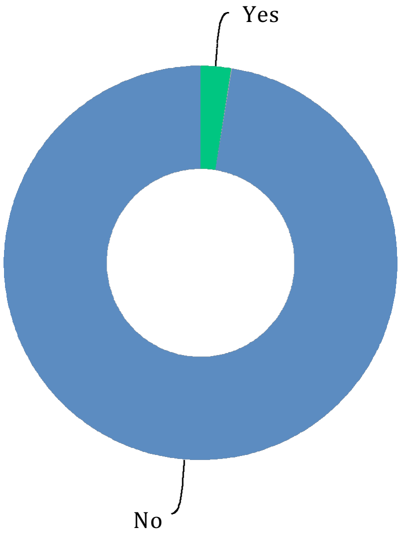

ANSWER CHOICES	RESPONSES	
Yes	2.51%	6
No	97.49%	233
TOTAL		239

Q21 If the answer was yes, is it because of you directly?

Answered: 191 Skipped: 49

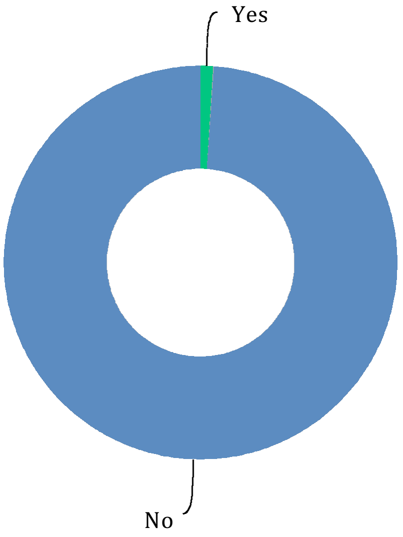

ANSWER CHOICES	RESPONSES	
Yes	1.05%	2
No	98.95%	189
TOTAL		191

Q22 Do you feel safe and protected?

Answered: 240 Skipped: 0

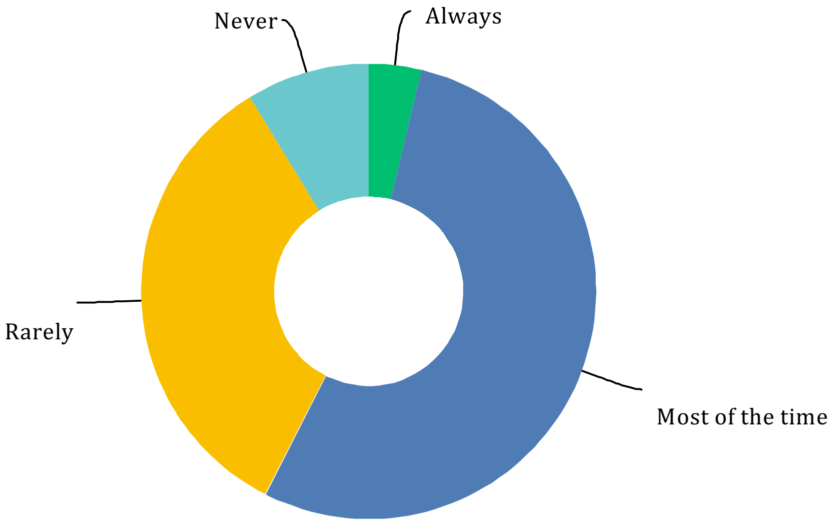

ANSWER CHOICES	RESPONSES	
Always	3.75%	9
Most of the time	53.75%	129
Rarely	33.75%	81
Never	8.75%	21
TOTAL		240

Q23 Do you feel that your family is safe?

Answered: 238 Skipped: 2

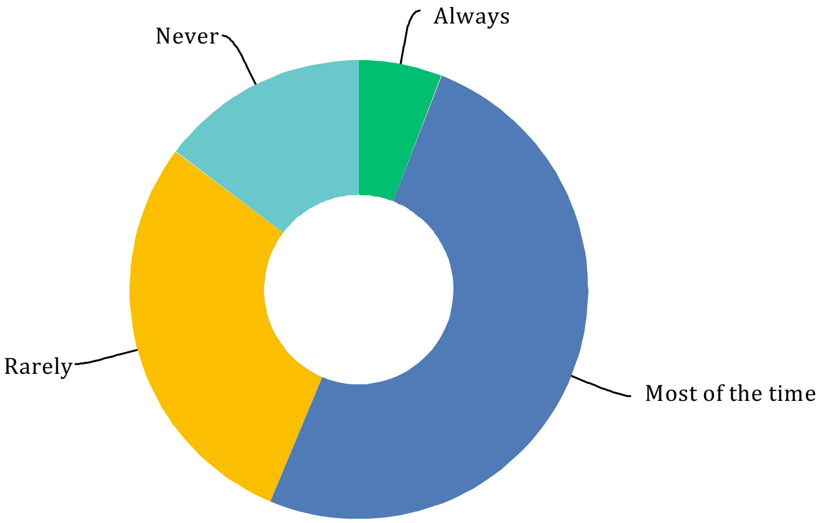

ANSWER CHOICES	RESPONSES	
Always	5.88%	14
Most of the time	50.42%	120
Rarely	28.99%	69
Never	14.71%	35
TOTAL		238

Q24 Are you maintaining good life style regarding your health?

Answered: 239 Skipped: 1

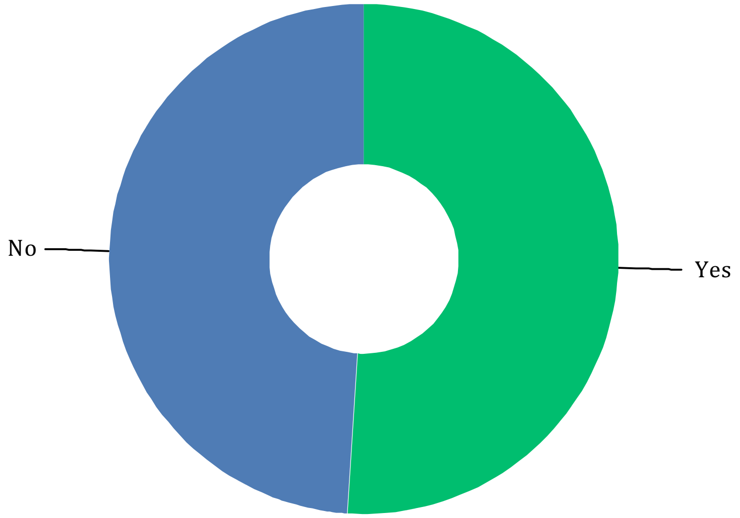

ANSWER CHOICES	RESPONSES	
Yes	51.05%	122
No	48.95%	117
TOTAL		239

Q25 Are you maintaining good life style regarding your food?

Answered: 239 Skipped: 1

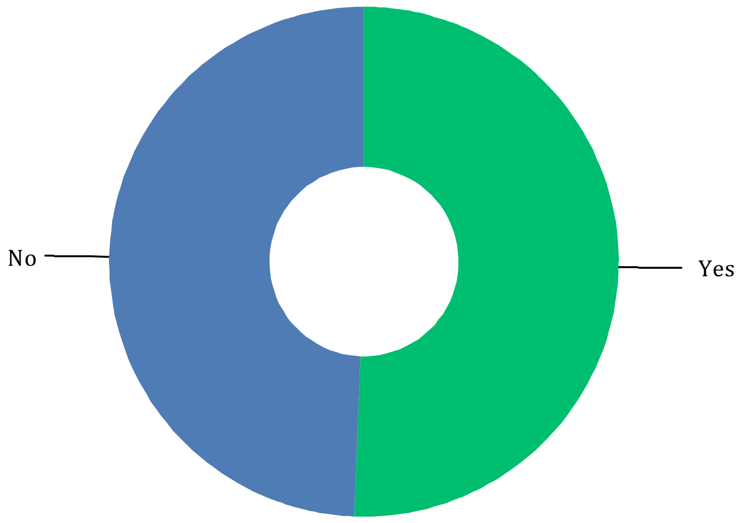

ANSWER CHOICES	RESPONSES	
Yes	50.63%	121
No	49.37%	118
TOTAL		239

Q26 Are you maintaining good life style regarding your sleep?

Answered: 239 Skipped: 1

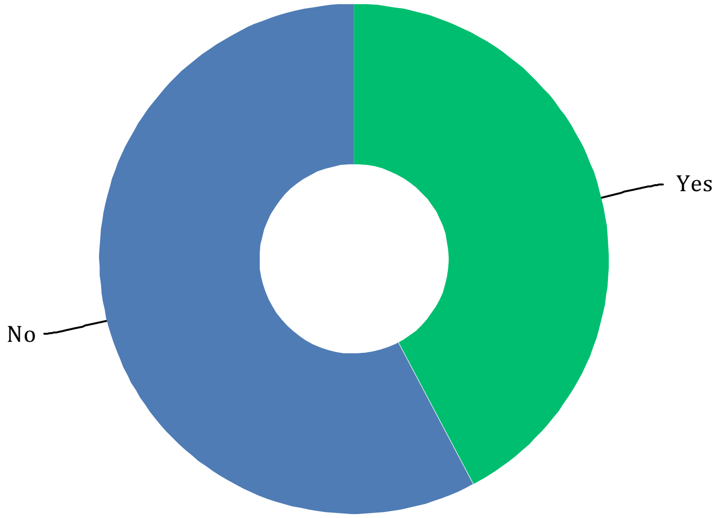

ANSWER CHOICES	RESPONSES	
Yes	42.26%	101
No	57.74%	138
TOTAL		239

Q27 Are you maintaining good life style regarding your exercise?

Answered: 240 Skipped: 0

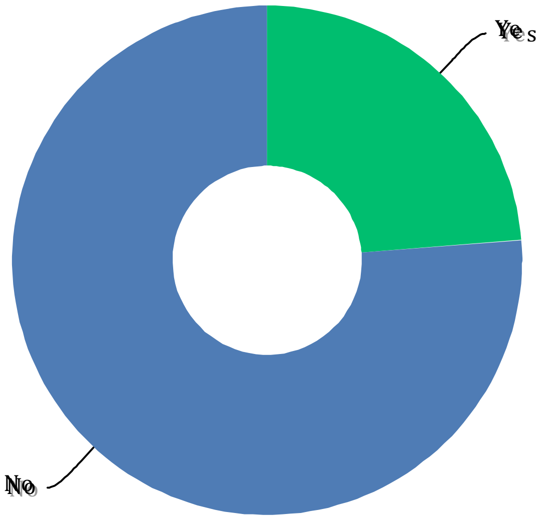

ANSWER CHOICES	RESPONSES	
Yes	23.75%	57
No	76.25%	183
TOTAL		240

Q28 Are you away from family?

Answered: 240 Skipped: 0

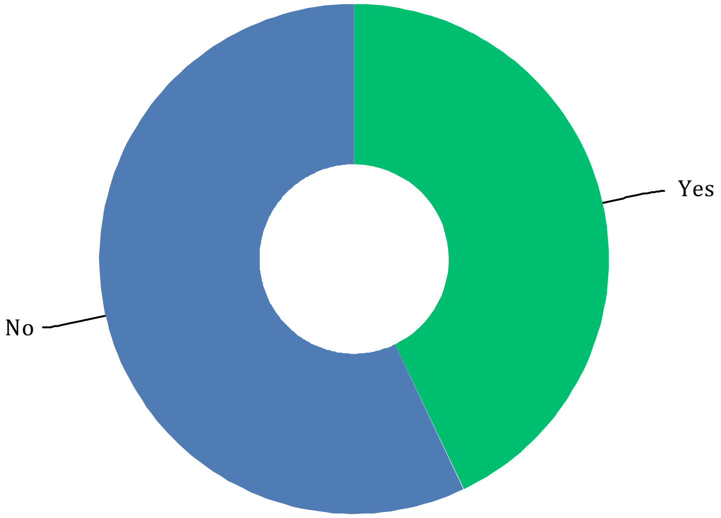

ANSWER CHOICES	RESPONSES	
Yes	42.92%	103
No	57.08%	137
TOTAL		240

Q29 Did you change your residence to protect your family?

Answered: 239 Skipped: 1

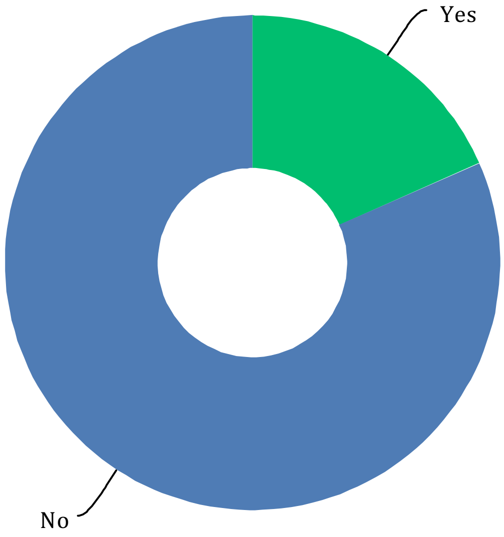

ANSWER CHOICES	RESPONSES	
Yes	18.41%	44
No	81.59%	195
TOTAL		239

Q30 Did you miss an exam during the pandemic?

Answered: 239 Skipped: 1

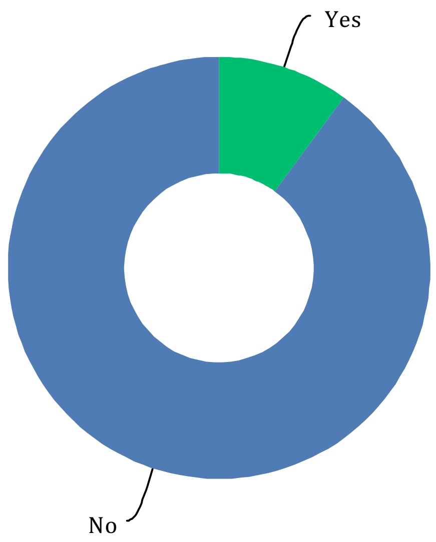

ANSWER CHOICES	RESPONSES	
Yes	10.04%	24
No	89.96%	215
TOTAL		239

Q31 Is there a reduction in the training activities during COVID-19 pandemic?

Answered: 240 Skipped: 0

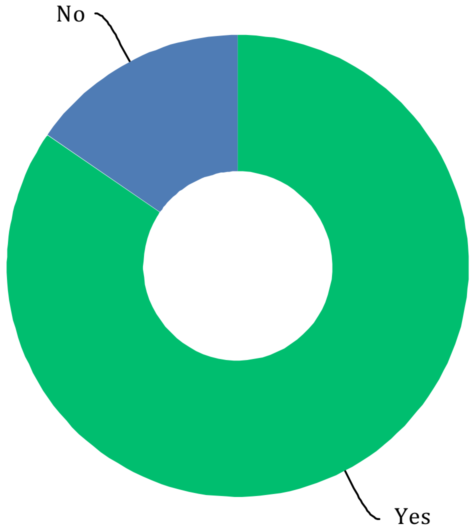

ANSWER CHOICES	RESPONSES	
Yes	84.58%	203
No	15.42%	37
TOTAL		240

Q32 For surgical specialties, is there a reduction in the level of surgical exposure and the number of operations during COVID-19 pandemic?

Answered: 179 Skipped: 61

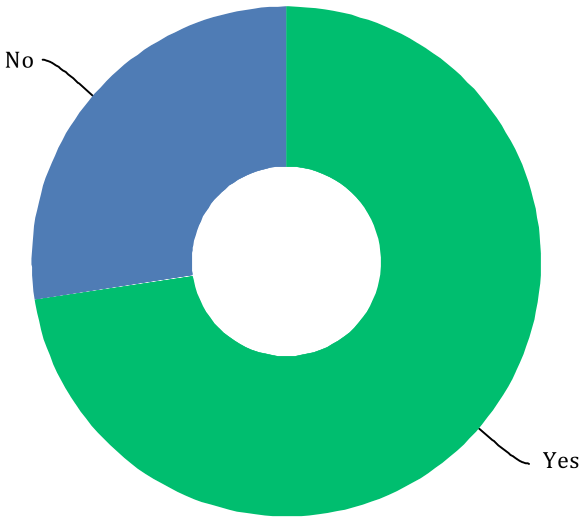

ANSWER CHOICES	RESPONSES	
Yes	72.63%	130
No	27.37%	49
TOTAL		179

Q33 Do you have enough time to read and study during the pandemic?

Answered: 239 Skipped: 1

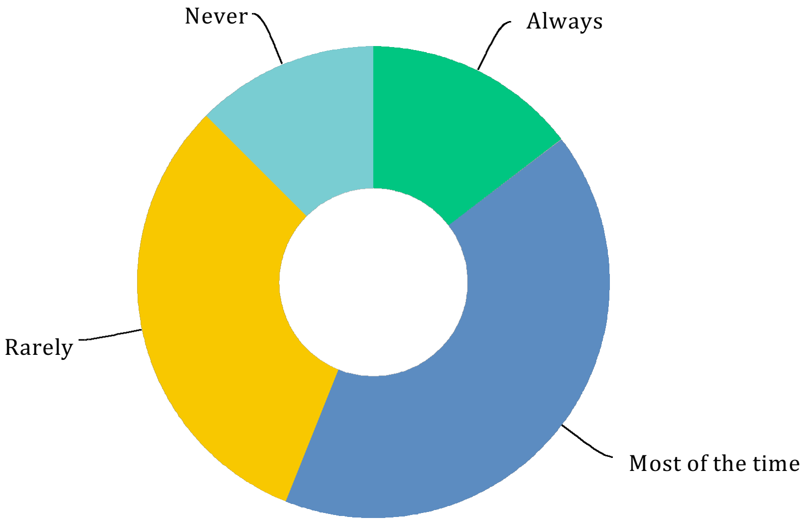

ANSWER CHOICES	RESPONSES	
Always	14.64%	35
Most of the time	41.42%	99
Rarely	31.38%	75
Never	12.55%	30
TOTAL		239

Q34 Do you feel that you are psychologically prepared to study and read?

Answered: 239 Skipped: 1

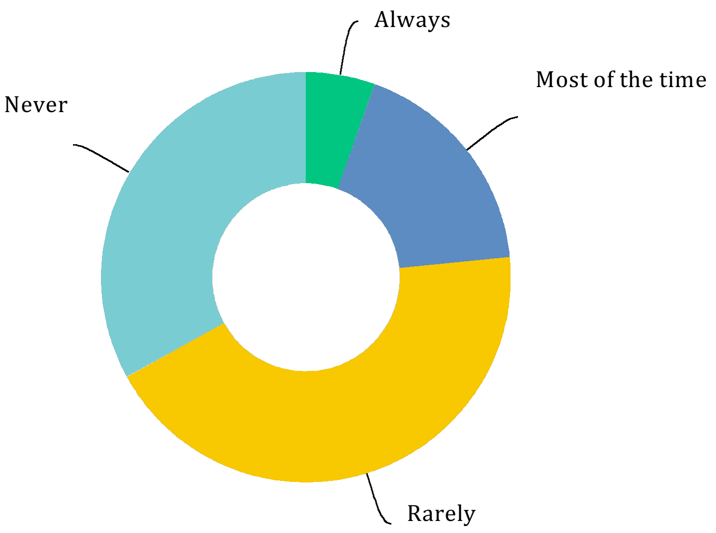

ANSWER CHOICES	RESPONSES	
Always	5.44%	13
Most of the time	17.99%	43
Rarely	43.51%	104
Never	33.05%	79
TOTAL		239

Q35 Do you feel stress because of the upcoming exams during the pandemic?

Answered: 240 Skipped: 0

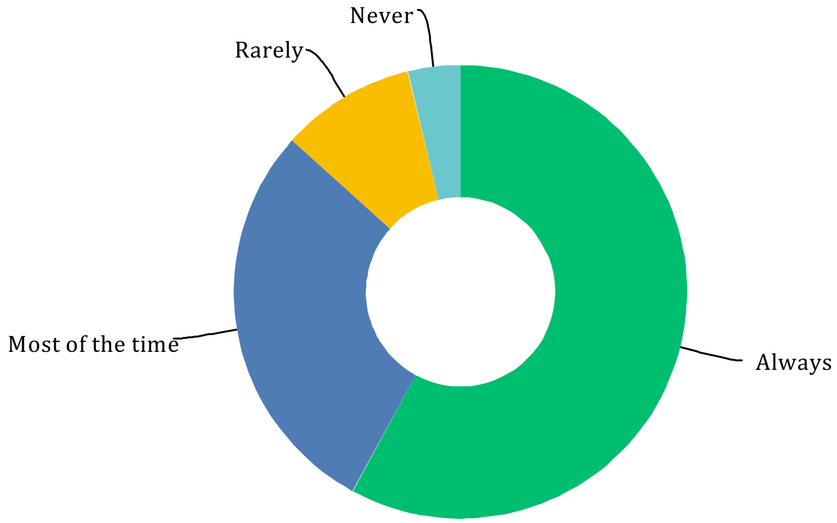

ANSWER CHOICES	RESPONSES	
Always	57.92%	139
Most of the time	28.75%	69
Rarely	9.58%	23
Never	3.75%	9
TOTAL		240
